# Hip, hip, hooray!

**DOI:** 10.7554/eLife.00646

**Published:** 2013-03-12

**Authors:** Emma Pewsey

**Affiliations:** Department of Materials Science and Metallurgy, University of Cambridge, Cambridge, United Kingdomep320@cam.ac.uk

**Keywords:** Science writing competition, outreach, open access

## Abstract

X-rays are best known for showing where bones have fractured, but researchers can also use X-rays to investigate why bones break, which could lead to treatments that reduce the number of elderly people who suffer broken hips.

This article by Emma Pewsey (pictured) was the winning entry in the Access to Understanding science-writing competition for PhD students and early career post-doctoral researchers organized by Europe PubMed Central in partnership with The British Library. Entrants were asked to explain to a non-scientific audience, in fewer than 800 words, the research reported in a scientific article and why it mattered.

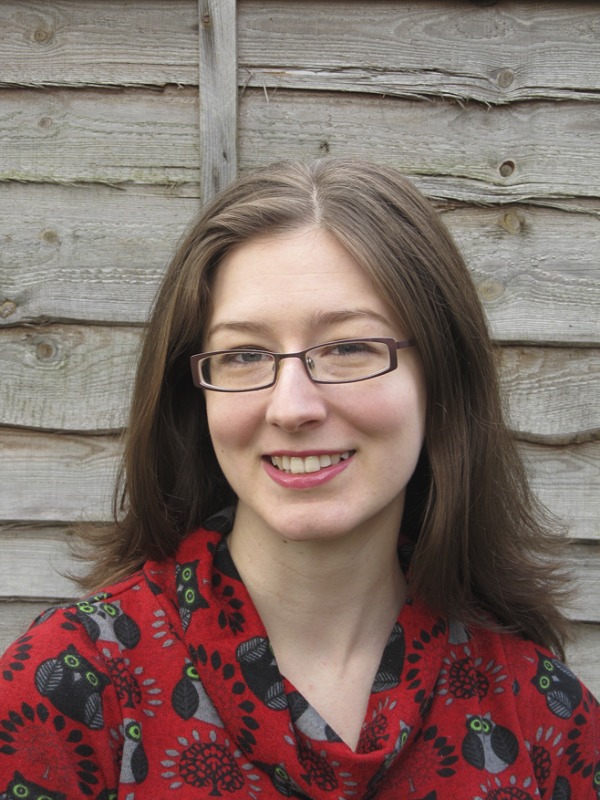

Normal healthy bones can be thought of as nature's scaffold poles. The tightly packed minerals that make up the cortical bone form a sheath around an inner core of spongy bone and provide the strength that supports our bodies. Throughout our lives, our skeletons are kept strong by the continuous creation of new, fresh bone and the destruction of old, worn out bone. Unfortunately, as we become older, destruction becomes faster than creation, and so the cortical layer thins, causing the bone to weaken and break more easily. In severe cases, this is known as osteoporosis. As a result, simple trips or falls that would only bruise a younger person can cause serious fractures in the elderly. However, half of the elderly patients admitted to hospital with a broken hip do not suffer from osteoporosis.

So why do those hips break? This is an important question because hip fractures are very debilitating. Repairing a fractured hip requires traumatic surgery that, even if successful, may not enable a patient to regain the full mobility they had beforehand. The National Osteoporosis Society estimates that 13,800 people in the UK die every year as a direct result of hip fractures. This is over 10% of patients injured (NHS Choices). Therefore, understanding how these fractures occur and acting to prevent them is vital for improving the quality and length of life of our aging population.

To better understand why hips fracture, researchers in Cambridge and Prague analysed CT scans performed on the opposite, unbroken hips of a group of elderly women admitted to Bulovka University Hospital in Prague with hip fractures (Poole et al., 2012). Previous studies have shown that these unbroken hips tend to be in a similar condition to the patient's other hip before it was fractured.

CT scanners are now standard pieces of equipment in most hospitals, and are used to examine organs and tissues inside the body. Essentially a rotating X-ray machine, a CT scanner takes many X-ray snapshots at different angles around a body part to produce a 3D image of its internal structure. X-rays are electromagnetic waves that are partially absorbed by the materials they pass through. The amount of absorption depends on the density of the structure encountered—denser structures, like bone, absorb more of the X-ray beam, leaving less to be measured by the detector on the other side. However, the resolution of the images collected by a standard hospital CT scanner is not sensitive enough to accurately determine the thickness of the cortical bone.

A new image processing technique has changed that. Using this technique, the researchers from Prague and Cambridge were able to extract information from clinical CT scans that was sensitive enough to produce false-coloured maps showing variations in the thickness of cortical bone in more detail than ever. Variations in thickness of only 30 microns—the size of a grain of dust—could be detected.

The results were striking. Not only did the women with fractured hips have generally thinner cortical bone than women in a control group, some of them also had local patches of even thinner bone. This was the case even in women who did not suffer from osteoporosis. Most importantly, the extra-thin regions were found on the femoral neck—the part of the hip bone where fractures most commonly occur. In some patients, these patches were 30% thinner than the surrounding bone, and as big as a thumbnail. These weaker points provide the ideal conditions for a crack to form and subsequently grow into a fracture. Further studies are needed to confirm whether these localised regions do act as the starting point for a fracture, but at the very least they affect the type, and hence severity, of fracture that occurs. These studies might also be able to explain why some hips fracture for no obvious reason. These spontaneous hip fractures account for 6% of all hip fractures, or 4000 broken hips every year in the UK.

These studies might also be able to explain why some hips fracture for no obvious reason.

The research team have named these local patches of thinner bone ‘focal osteoporosis’. However, despite the name, it is not yet known if these areas can be strengthened using standard osteoporosis drugs, which slow down the natural destruction of bone cells. An extensive clinical trial will be needed to investigate further, but if the focal patches do respond to treatment, it raises the tantalising possibility of a future where many fractures could be treated before they even form. The improvement this would have on our quality of life in old age would be invaluable.

## References

[bib1] NHS Choices Hip fracture. http://www.nhs.uk/conditions/hip-fracture/Pages/introduction.aspx.

[bib2] Poole KES, Treece GM, Mayhew PM, Vaculik J, Dungi P, Horák M (2012). Cortical thickness mapping to identify focal osteoporosis in patients with hip fracture. PLOS One.

